# Classification of Polar Maps from Cardiac Perfusion Imaging with Graph-Convolutional Neural Networks

**DOI:** 10.1038/s41598-019-43951-8

**Published:** 2019-05-20

**Authors:** Nathalia Spier, Stephan Nekolla, Christian Rupprecht, Mona Mustafa, Nassir Navab, Maximilian Baust

**Affiliations:** 1 Computer Aided Medical Procedures & Augmented Reality, Faculty of Informatics, Technical University of Munich, Munich, Germany; 2 Nuklearmedizinische Klinik und Poliklinik, Klinikum rechts der Isar, Technical University of Munich, Munich, Germany

**Keywords:** Cardiology, Information technology, Scientific data, Software

## Abstract

Myocardial perfusion imaging is a non-invasive imaging technique commonly used for the diagnosis of Coronary Artery Disease and is based on the injection of radiopharmaceutical tracers into the blood stream. The patient’s heart is imaged while at rest and under stress in order to determine its capacity to react to the imposed challenge. Assessment of imaging data is commonly performed by visual inspection of polar maps showing the tracer uptake in a compact, two-dimensional representation of the left ventricle. This article presents a method for automatic classification of polar maps based on graph convolutional neural networks. Furthermore, it evaluates how well localization techniques developed for standard convolutional neural networks can be used for the localization of pathological segments with respect to clinically relevant areas. The method is evaluated using 946 labeled datasets and compared quantitatively to three other neural-network-based methods. The proposed model achieves an agreement with the human observer on 89.3% of rest test polar maps and on 91.1% of stress test polar maps. Localization performed on a fine 17-segment division of the polar maps achieves an agreement of 83.1% with the human observer, while localization on a coarse 3-segment division based on the vessel beds of the left ventricle has an agreement of 78.8% with the human observer. Our method could thus assist the decision-making process of physicians when analyzing polar map data obtained from myocardial perfusion images.

## Introduction

### Clinical background

Myocardial Perfusion Imaging (MPI) is a non-invasive imaging modality commonly used in the diagnosis of Coronary Artery Disease (CAD)^1^, where the uptake of the injected radiopharmaceutical can be measured using positron emission tomography (PET) or single photon emission computed tomography (SPECT) and is related to the myocardial perfusion. In order to be able to identify the regions with reduced perfusion, the patient’s heart is imaged twice, once at rest and once under stress. Visual interpretation of the images is dependent on user experience, and thus quantitative data is also routinely incorporated into the evaluation as suggested by the American Society of Nuclear Cardiology (ASNC)^2^.

**Figure 1:**
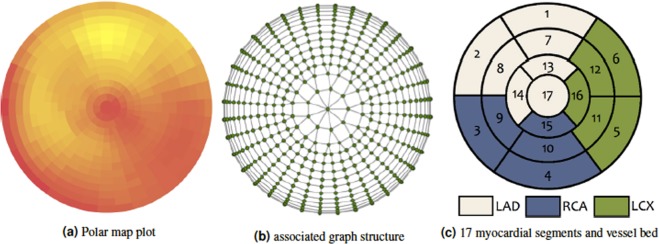
(**a**) Polar map derived from MPI. (**b**) Graph encoding the spatial relationship of the intensity values of the polar map depicted in (**a**). (**c**) Anatomical division showing to which major vessel bed each myocardial segment is assigned to. Adapted from *Cerqueira et al*.^23^.

Quantitative perfusion parameters are extracted from MPI and displayed as a polar map (Fig. 1a). Moreover, quantitative analysis of myocardial perfusion imaging data usually requires the comparison to normal limits, which are obtained from groups of patients retrospectively identified as normal. A common assumption for most databases is that the intensity values at each location follow a Gaussian distribution, which might not always be the case^3^. Furthermore, it has been shown^4^ that physicians improve their diagnostic performance when assisted by an automated CAD detection system based on polar map images. Towards this end, we propose a deep-learning-based method that does not rely on normal databases to automatically detect and localise CAD on polar maps and that could be used in a clinical setting to assist the decision-making process of physicians when analysing polar map data obtained from MPI, e.g. by providing a second opinion.

### Related work

*Fujita et al*.^5^ used a multilayer perceptron with 16 × 16 input nodes, 100 hidden nodes and eight output nodes to classify digitized and down-sampled polar maps into normal or pathological with respect to one of the following areas: Left Circumflex Artery (LCX), Right Coronary Artery (RCA) and Left Anterior Descending Artery (LAD) as well as combinations thereof (LAD + LCX, LCX + RCA, LAD + RCA, LAD + LCX + RCA). The segmentation of the polar maps into these areas is depicted in Fig. 1c. This approach was trained and evaluated using 74 cases examined by coronary angiography and it has been reported that the achieved recognition performance was better than that of a radiology resident, but worse than the one of the participating experienced radiologist. *Porenta et al*.^6^ used a similar network architecture with 45 input nodes (corresponding to the relative segmental thallium uptake at stress), 15 hidden nodes and one output node indicating the presence of CAD. This study was conducted using 159 patients, with coronary angiography being available for 81 patients, and the network’s average sensitivity was reported to be 51% (at a specificity of 90%) in comparison to 72% achieved by an expert reader. Also similar to *Fujita et al*.^5^, *Hamilton et al*.^7^ used an artificial neural network with 15 × 40 input nodes, 5 hidden nodes and one output node. This study was conducted using both simulation data and real stress-rest data obtained from 410 male patients. The accuracy of the network for detecting CAD was reported to be 92%. *Lindahl et al*.^8^ conducted a conceptually similar study using 135 patients, where a contrast left ventriculogram was performed in 106 cases. This study particularly investigated the usage of quantization-based and Fourier-transform-based dimensionality reduction techniques for reducing the input data size. The authors also trained several networks for detecting CAD, CAD in the LAD territory, and CAD in the RCA/LCX territory. They reported a statistically significant improvement of over 10% in terms of sensitivity in comparison to two human experts for the detection of CAD. All these methods have in common that they directly use quantized and down-sampled versions of the original polar maps as an input to a 3-layer neural network (or 3-layer-perceptron) with one hidden layer. Hence, we term these approaches *direct methods*.

In contrast to this, there also exist several *indirect methods* which aim for a computer-assisted diagnosis of CAD via image-derived quantitative measures that are used for classification. An early example is the work of *Slomka et al*.^9^, who proposed a method based on intensity-based image registration and normalization in order to derive a relative count change measure, i.e. the measure of ischemia (ISCH). ISCH was reported to significantly outperform existing quantitative approaches based on reference databases. *Asanjani et al*.^10^ extended this concept and proposed a method based on support vector machines taking ISCH and Stress Total Perfusion Deficit (TPD) as well as functional parameters, such as Poststress Ejection Fraction Changes or Motion and Thickening Changes, as a classifier input. In a related work, *Asanjani et al*.^11^ used even more inputs, i.e. supine/prone TPD, stress/rest perfusion changes, transient ischemic dilatation, age, sex, and post-electrocardiogram CAD probability, as an input to a boosted ensemble classifier. The diagnostic accuracy of the classifier in the latter study has been shown to be on par or slightly better than the one achieved by two participating experts, respectively^11^.

Recently, *Betancour et al*.^12^ proposed a hybrid method based on deep convolutional neural networks which takes into account both raw and quantitative (based on TPD) polar maps for the prediction of obstructive stenosis. The approach has been developed using data from 1,638 patients. Using a threshold to match the specificity of TPD, per-patient sensitivity has been reported to improve from 79.8% (TPD) to 82.3% (*p* < 0.05), and per-vessel sensitivity to improve from 64.4% (TPD) to 69.8% (*p* < 0.01). In a subsequent multi-center study, *Betancour* *et al*.^13^ applied a comparable method to predict obstructive CAD taking into account both upright and supine polar maps. The authors showed that when operating with the same specificity as clinical readers, the proposed method had the same sensitivity for disease prediction as on-site clinical readers, and significantly improved the sensitivity compared to combined total perfusion deficit (cTPD).

### Contributions

In this study, we evaluate the potential of Graph Convolutional Neural Networks^14,15^ (GCNNs) for the task of classifying polar maps obtained from MPI, cf. Fig. 1a, into normal and abnormal. As these polar maps are images with intensity values being arranged in a polar grid, GCNNs provide a natural way of applying convolutional neural networks to such maps without the need of re-sampling them w. r. t. a rectangular grid. By associating a graph structure with the (polar) image data, cf. Fig. 1b, convolutions can be generalized to arbitrary arrangements of data and the MPI-derived polar maps can be directly fed to convolutional neural networks, that are based on these generalized convolution operations. In contrast to this, previous works utilizing neural networks^5–8,12^ require the re-sampling of polar maps w. r. t. a standard rectangular image grid. Another important difference to the recent work of *Betancour et al*.^12^ is the fact that the proposed method takes only the polar maps as an input and no additional data.

The main goal of this study is to investigate whether, and the extent to which, the knowledge of an expert reader assessing these polar maps can be acquired by such networks. In addition to this, we also investigate whether attribution techniques for visualizing image parts that are responsible for a certain decision of the classifier^16^ can be used for disease localization. However, the latter task is not the main goal of this work and thus only evaluated on 30 polar maps.

## Results

We evaluate the proposed approach with respect to classification of polar maps into normal and abnormal and the localization of pathological areas separately. For CAD classification we evaluate two GCNNs, i.e. one based on Chebyshev polynomials and one based on Cayley polynomials, and a Fully Connected Network (FCN) as well as a Convolutional Neural Network (CNN) as baseline methods.

### Classification results

At first, we evaluated the models w. r. t. their ability of classifying a given polar map into abnormal (presence of CAD) or normal regardless of localization. In order to fully utilize the dataset and validate the performance of the models, we performed 4-fold cross-validation, cf. Table 1. For each selected fold, the three remaining folds were used for training. A consolidated view on these experiments is shown in Table 2. The proposed GCNN model using Chebyshev filters achieved the highest performance of detection for both rest and stress tests showing an agreement with the human observer of 89.3% and 91.2% respectively.

**Table 1:** Results of 4-fold cross-validation.

	fold 1	fold 2	fold 3	fold 4
Total number of patients (rest/stress)	125/110	125/110	125/111	127/112
Abnormal cases (rest/stress)	59/51	59/51	59/52	60/52
FCN acc. rest/stress	77.8/84.7	80.1/83.8	88.1/81.1	91.2/83.6
CNN acc. rest/stress	84.1/88.3	85.7/81.1	85.7/73.0	79.2/89.1
GCNN Chebyshev acc. rest/stress	84.7/91.9	84.9/91.9	90.5/90.9	96.0/90.0
GCNN Cayley acc. rest/stress	84.0/92.0	87.2/90.0	90.4/91.0	93.6/90.0

Please note that classifiers for classifying abnormal cases have been trained separately for rest and stress cases. Evaluation results show average agreement with the human observer (measured in percent) for the respective fold when trained on the three remaining folds.

**Table 2:** Classification performance.

Model	Rest Classifier			Stress Classifier			Trainable Parameters
	Agreement	Sensitivity	Specificity	Agreement	Sensitivity	Specificity	
FCN	84.3	80.5	87.0	83.3	71.4	93.8	212,982
CNN	83.7	76.8	90.0	82.9	65.8	**98.0**	12,462,594
GCNN Chebyshev	**89.3**	85.1	**92.8**	**91.2**	**85.7**	95.9	12,399,730
GCNN Cayley	88.8	**86.9**	90.0	90.8	84.3	95.8	5,9999,204

Best results are shown in bold and all values are in percentage of agreement with the human observer.

### Localization results

**Figure 2:**
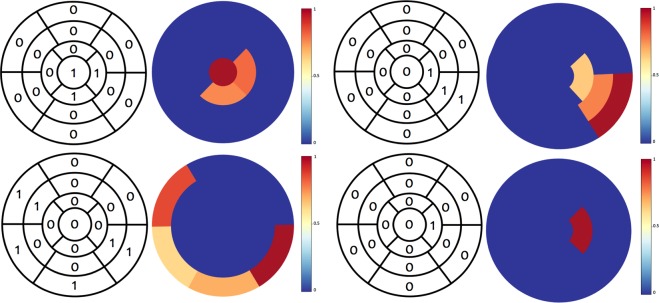
Classification of myocardial segments w.r.t. to abnormality. The color corresponds to the probability of the respective segment being pathological, ranging from 0 (blue, normal) to 1 (red, abnormal).

We assessed the ability to localize the presence of CAD on a 17-segment division and on a coarse 3-segment division which is based on coronary artery blood supply to the finer segments as shown in Fig. 1c. For assessing localization performance, the model that agreed best with the human observer, i.e. GCNN Chebyshev, was selected. Next, we used 30 abnormal polar maps randomly drawn from the whole data set and trained the model on the remaining abnormal data. In Fig. 2 the localization prediction from the model for four different polar maps have been shown. As presented in Table 3, the model has a localization sensitivity of 47.2% at a specificity of 90.7%, if carried out on a 17-segment division. If performed on a coarser division, localization sensitivity increases to 83.2% at a specificity of 70.8%. A coarser localization, performed on a “per vessel“ basis, reflects the analysis clinicians would normally perform. Thus, Table 4 shows the model’s performance for each of the 3 main vessel beds individually. Localization is best in the RCA region and worst in the LAD region with a human observer agreement of 95.4% and 63.6%, respectively.

**Table 3:** General localization results for 17 and 3-region segments.

Number of Segments	Agreement	Sensitivity	Specificity
17	83.1	47.2	94.4
3	78.8	83.2	70.8

**Table 4:** Localization performance on each of the main vessels beds.

Main Vessel	Agreement	Sensitivity	Specificity
RCA	95.4	100	85.7
LAD	63.6	50.0	71.4
LCX	77.3	84.2	33.3

## Discussion

**Figure 3:**
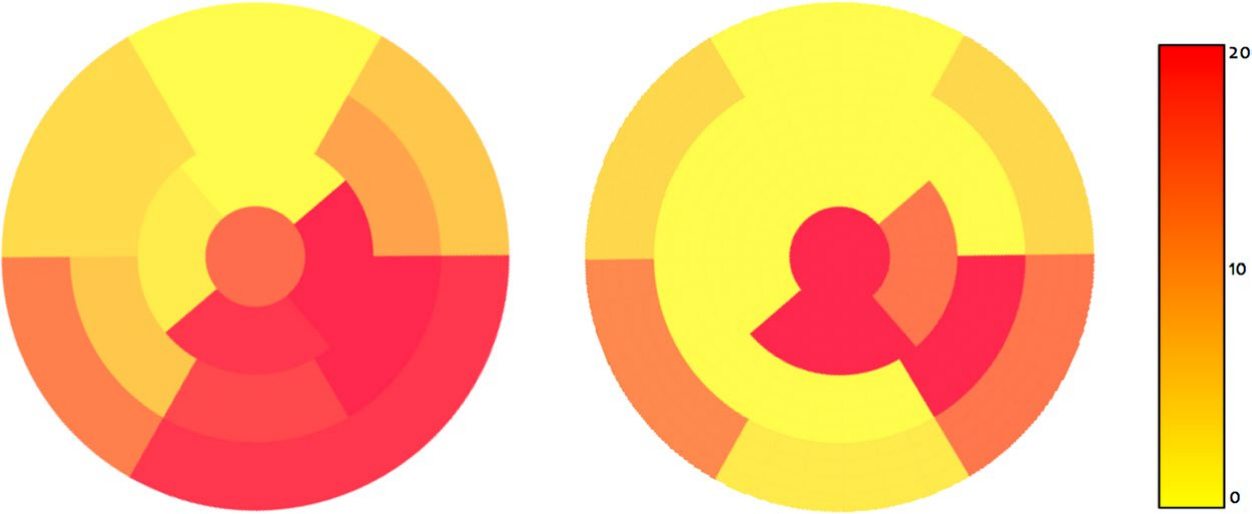
Heatmaps indicating the frequency of annotated pathological segments (left) and the frequency of segments being detected as pathological. Color bar indicates frequency of detections in a particular region: high frequency - red, low frequency - yellow.

In this study, we propose to use GCNN for the classification and localization of CAD on myocardial polar maps. First, we assess the model’s classification performance by comparing it to two baseline methods and we are able to show significant improvements w.r.t. agreement using GCNN with Chebyshev filters. Localization performance is evaluated using 30 abnormal polar maps randomly drawn from the whole data base and using the best performing architecture, i.e. GCNN Chebyshev, trained on the remaining abnormal data. These polar maps have been labeled on a segment basis by an expert. The network has a higher localization agreement when using a fine division, albeit at the cost of very low sensitivity. Using the more clinically relevant coarse division (into LAD, LCX, and RCA), the network’s sensitivity increases while still maintaining similar agreement levels. However, the localization performance of the model is better in the RCA and LCX regions than in the LAD region. Figure 3 shows a heat map of accumulated abnormalities present in different regions amongst all 30 ground truth images and a heat map of all abnormal detections amongst all 30 localization experiments. The heatmap in the left panel of Fig. 3 shows that abnormalities are predominant in the RCA and LCX regions - at least within the 30 polar maps used for evaluation. The poor localization performance of the models could thus be caused by a lack of training data with abnormalities in the LAD territory. Furthermore, another very likely reason for this finding could be the camera itself. While some centers perform the MPI scans on a D-SPECT in supine and prone position, this study used only data from a sitting upright position. Nonetheless, The model’s localization performance is comparable to that of previous studies which use separate networks to identify the presence of CAD in each of the main vessel beds.

As our evaluation focuses on the agreement of the proposed network architecture with the expert reader, we performed an inter-observer study on the 30 polar maps used for assessing the localization performance using the annotations from a second reader. The inter-observer variability on the 17-segment division has a Cohen’s kappa value of 0.79. The variability has a kappa value of 0.87 when evaluated on the 3-segment division, indicating a substantial agreement between the two readers.

These findings suggest that the proposed model could be an attractive alternative to the conventional analysis of polar maps using normal databases in order to further support the clinical decision-making process by providing an automated second opinion.

One of the advantages of using GCNNs for analyzing polar maps from cardiac perfusion imaging is that they can directly operate on the polar maps themselves. This way, GCNNs do not require any re-sampling of the polar maps w.r.t. a rectangular grid, which could potentially increase the number of input parameters. Enforcing a two-dimensional CNN to use an equal amount of input parameters and as well as approximately the same number of trainable parameters yields considerably worse results as shown in Table 2 (cf. second and third row). Furthermore, areas relevant for the classification decision can be directly visualized on the polar map data using standard attribution techniques^16^ as shown in Fig. 2.

Despite the promising results, our study has several limitations. Localization performance was evaluated against a small number of manually segmented polar maps. Furthermore, the model’s performance was not assessed against coronary angiography ground truth, but only on data labeled by an expert reader, who did not have access to other clinical information. While this ensures that the reader was not biased by aspects such as the patient’s smoking history, it does not correspond to the regular clinical reading scenario. Also, a comparison with a conventional normal database has not been performed in this work. Moreover, polar maps were obtained from one particular camera system and further evaluation from other centers with different cameras would likely be required. In addition to this, it deserves to be mentioned that generalizing the achieved results to other data bases might be harder than in the case of normal limits.

## Methods

This section describes our GCNN-based approach to polar map detection and localization of CAD. This approach is more suited to the unstructured nature of polar maps. We compare the proposed method and discuss its advantage over two baseline methods. These techniques are chosen to provide a competitive baseline while representing a basic approach to the problem, thus, a fully connected neural network and a shallow convolution neural network were chosen. While more complex network structures could be used, they would run a greater risk of over-fitting. Each polar map comprises of 460 nodes and we denote a training sample as (*x*_*i*_, *y*_*i*_) where ${x}_{i}\,\in \,{{\mathbb{R}}}^{460}$ is a vector representing the *i*^*th*^ sample, and *y*_*i*_ ∈ {0, 1} is the corresponding label indicating a polar map is normal or pathological respectively.

Two polar maps are generated for every patient and their individual valuation is taken into account when interpreting the patient’s overall outcome. Thus, we decided to train networks for two tasks: rest classification, i.e. assess whether rest test is abnormal or not, and stress classification, i.e. assess whether stress test is abnormal or not.

### Data acquisition

The dataset consists of 946 labeled polar maps, 503 of which are rest tests and the remaining stress tests. Amongst rest tests, 237 are labeled as abnormal and 266 as normal. For stress tests, 206 are labeled abnormal and 237 normal. The samples were obtained from the Nuclear Medicine Department at Klinikum Rechts der Isar, Munich, Germany, using a dedicated SPECT imaging system (D-SPECT, Spectrum Dynamics, Caesarea, Israel) with a one-day protocol and patients in an upright sitting position. The abnormal cases have been selected on a consecutive basis in 2017. The normal cases were identified in a three reader consensus decision from data acquired in 2014 and 2015. The readers had access only to the polar maps and were blinded to any other information. This setup was used as the investigated algorithm had only access to the polar maps as well. Image acquisition protocols are based on *Nakazato et al*.^17^ and *Nudi et al*.^18^. The polar maps were generated using the in-house developed analysis software MunichHeart^19^. As a pre-processing step, each polar map is normalized by its highest perfusion value. Data augmentation is not carried out.

### 1D Fully connected neural network

First, we examine the conventional approach. We vectorized the original polar map and used the plain 460-dimensional vector of polar map intensities as an input to the a fully connected 3-layer neural network with 460 hidden nodes and two output nodes. This approach is comparable to previous related works^5–7^, but uses the full information of the polar map without sub-sampling or quantization of intensity values. Here, the fully connected layers ensure that the spatial arrangement is implicitly learned. A Rectified Linear Unit (ReLU) activation function is used for the neurons in the hidden layer and a softmax activation function is used for the output layer. Categorical cross entropy is used as the loss function.

### 2D Convolutional neural network

**Figure 4:**
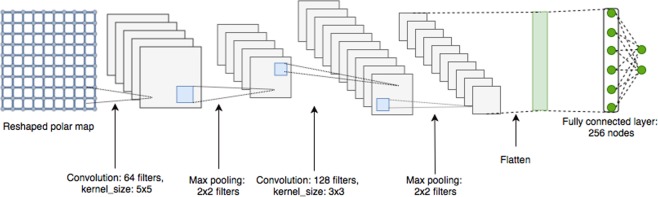
Convolutional Neural Network Architecture.The network’s input is a polar map reshaped to size 23 × 20. We use two convolution layers, each followed by max pooling. The first layer contains 64 filters of size 5 × 5 and the second layer contains 128 filters of size 3 × 3. The output is preceded by a fully connected layer of size 256.

In this model, we create a convolutional network by reshaping the polar map into a 2D array of size 23 by 20. The rationale being that small filters, e.g. 5 × 5, could learn the relationship between the individual entries of the vector. Our CNN architecture employs only 2 layers of convolutional filters with a kernel size of 5 × 5 in the first layer and 3 × 3 in the second. Each layer was followed by a ReLU activation function as well as a max-pooling layer with a 2 × 2 pixel window. The network ends with a fully-connected layer with a softmax, cf. Figure 4. Categorical cross entropy is used as the loss function.

### Graph-based convolutional neural network using chebyshev polynomials

**Figure 5:**
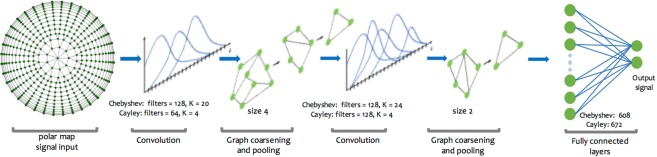
Graph Convolutional Neural Network Architecture. The network’s input is the the polar map data defined on a graph that encodes the neighborhood relationships between the individual data points. We use two layers of convolution filter each followed by graph coarsening and pooling layers. When training with Chebyshev polynomials we use filters of order 16 in the first layer and 32 in the second. Cayley polynomials of order 4 and 6 are used in the first and second layer respectively. Both models employ a pooling size of 4 in the first layer and 2 in the second.

In both previous baselines the spatial arrangement of the data is not taken into account. We address this problem by representing the polar map as an undirected graph and performing convolutional operations in the spectral domain. Here, a polar map is represented as a graph ${\mathscr{G}}=({\mathscr{V}},\, {\mathcal E} ,\,A)$ where ${\mathscr{V}}$ is the set of 460 vertices, ${\mathcal E}$ is the set of edges and $A\in {{\boldsymbol{{\mathbb{R}}}}}^{460\times 460}$ is the adjacency matrix representing the weight of each edge. We assume that every edge has the same weight. The graph Laplacian is then defined as *L* = *D* − *A*, where $L\in {{\mathbb{R}}}^{460\times 460}$ and $D\in {{\mathbb{R}}}^{460\times 460}$ is the diagonal degree matrix $D=diag({\sum }_{i\ne j}\,{A}_{ij})$. Since the Laplacian is symmetric and positive semi-definite, we can decompose it as *L* = *U* Λ *U*^*T*^ where the columns of $U\in {{\mathbb{R}}}^{460\times 460}$ are the set of orthonormal eigenvectors and ${\rm{\Lambda }}\in {{\mathbb{R}}}^{460\times 460}$ is a diagonal matrix of their associated eigenvalues. The vector $x\in {{\mathbb{R}}}^{460}$ is then defined on the nodes of the graph where *x*_*i*_ is the value of *x* at the *i*^*th*^ node. We can then write the convolution between two graph signals as $x\,\ast \,z=U{g}_{\theta }({\rm{\Lambda }}){U}^{T}x$. As proposed by *Defferrad et al*.^20^, filters are represented using Chebyshev polynomials:

1 $${g}_{\theta }({\rm{\Lambda }})=\sum _{k=0}^{K-1}\,{\theta }_{k}{T}_{k}(\tilde{{\rm{\Lambda }}}),$$

where ${T}_{k}(\tilde{{\rm{\Lambda }}})\in {{\mathbb{R}}}^{460\times 460}$ represents a Chebyshev polynomial of order k applied to a frequency $\tilde{{\rm{\Lambda }}}\in [\,-\,\mathrm{1,1]}$. ${\theta }_{k}\in {{\mathbb{R}}}^{K}$ are the coefficients that parametrize the filter and that are optimized during training. These filters are used in order to obtain spectral localization, and computational complexity comparable to classical CNNs $({\mathscr{O}}(K| {\mathcal E} |))$. Pooling is done by coarsening the graph using Graclus^21^ algorithm and performing max pooling on the collapsed vertices. Our GCCN architecture, depicted in Fig. 5, consists of 2 convolutional layers, each followed by a pooling layer and ReLU activation function. Max-pooling of sizes 4 and 2 were used for the first and second layer respectively. The network is trained with a cross entropy loss function.

### Graph-based convolutional neural network using cayley filters

In this model, proposed by *Levie et al*.^22^, spectral filters are instead represented by a Cayley polynomial of order K:

2 $${g}_{c,h}({\rm{\Lambda }})={c}_{0}+2Re\{\sum _{k=1}^{K}\,{c}_{k}{(h{\rm{\Lambda }}-i)}^{k}{(h{\rm{\Lambda }}+i)}^{-k}\},$$

where the filter parameters are a vector, ${\bf{c}}=({c}_{0},\,\mathrm{...,}\,{c}_{K})$, with one real coefficient and K complex ones and *h*, the spectral zoom. While maintaining the same computational complexity (assuming a sparsely connected graph), these filters provide better localization in frequency by using *h* to focus on particular bands of frequency, which might be more important for the overall understanding of the underlying structure. Our model using Cayley filters also employs a 2-layered network, albeit with lower order polynomials (Fig. 5). Cross entropy was also used as the loss function.

### Localization technique

In addition to classifying rest and stress polar maps into normal and abnormal, we wanted to evaluate how well the trained networks can be used for the localization of pathological areas. To this end, 30 polar maps exhibiting pathologies were randomly drawn from the entire data base and subdivided into the 17 clinically relevant segments depicted in Fig. 1c. Next, a clinical expert labeled all segments of these polar maps as either pathological or normal. Finally, we trained the GCNN Chebyshev model on the remaining abnormal data. To use a trained network for CAD localization, we drew inspiration from attribution techniques introduced by *Zeiler et al*.^16^. We replaced all intensities corresponding to graph nodes within a specified segment by the average intensity value of all intensity values in this segment computed over all normal cases. This modified polar map was then fed to the network and the probability for this modified polar map being abnormal was recorded. We repeated this process for all 17 segments and created a heatmap representation via assigning the respective classification score to all nodes with a segment. This way, we obtained a heatmap of the same size as the original polar map, where each of the 17 segments shows the probability of this case being abnormal when replacing the intensity values in the respective segment with the segment-wise averages obtained from the normal cases, see also Fig. 2. Please note that the threshold for classifying a segment as pathological has been determined using exhaustive search and was set to 0.4.

### Implementation and training hardware

The networks were trained on a Linux-based system with 128 GB RAM, Intel(R) Xeon(R) CPU @ 3.50 GHz and a 12 GB GeForce GTX TITAN X graphics card. Graph-based models were implemented with TensorFlow. Baseline methods were implemented with Keras. All models have a learning rate of 1e-3 and are optimized with Stochastic Gradient Descent (SGD). Baseline methods are trained in mini batches of 64 samples for 70 epochs. Both graph-based methods run for 60 epochs, with Chebyshev model using a mini batch size of 30 samples and the Cayley model with a mini batch size of 20. Convergence was assessed by verifying that the categorical cross-entropy loss in all cases did not decrease after the aforementioned number of epochs.
